# Serum albumin adjusted calcium and fluoride among type 2 diabetes mellitus patients

**DOI:** 10.6026/973206300200065

**Published:** 2024-01-31

**Authors:** Shruti Divya Dubey, Shashidhar Kurpad Nagaraj, Harish Rangareddy

**Affiliations:** 1 Sri Devaraj Urs Medical College constituent of Sri Devaraj Urs Academy of Higher Education and Research, Kolar, India; 2 Department of Biochemistry, Sri Devaraj Urs Medical College constituent of Sri Devaraj Urs Academy of Higher Education and Research, Kolar, India; 3 Department of Biochemistry, Haveri Institute of Medical Sciences, Haveri, India

**Keywords:** minerals, homeostasis, diabetes mellitus

## Abstract

The regulation of glucose-dependent insulin secretion in pancreatic β-cells relies on calcium concentration, making it a calcium-dependent process. Disruptions in calcium homeostasis may contribute to insulin dysfunction and disturbances in glucose homeostasis, potentially playing a role in the development of type 2 diabetes mellitus (T2DM). In T2DM patients, there may be changes in fluoride metabolism due to impaired renal function. Measurement of serum albumin-adjusted calcium and is crucial since changes in albumin levels can impact the accurate estimation of true calcium.

## Background:

Type 2 Diabetes mellitus (T2DM) is a major metabolic disorder characterized by chronic state of hyper-glycemia and is linked to an increased risk of a variety of chronic conditions, including cardiovascular disease and nephropathy [1, 2]. T2DM has been identified as a major cause of global morbidity and mortality [2]. Altered serum calcium levels have been linked to a number of metabolic abnormalities, including an increased risk of diabetes [3]. The essential mineral calcium serves as a nearly ubiquitous intracellular messenger, regulating a wide array of cellular functions, including muscle contraction, gene transcription, cell proliferation and cell signalling [4]. In addition, calcium plays a key role in insulin secretion and glucose homeostasis. Glucose-dependent insulin secretion is a calcium regulated process dependent on intracellular calcium concentration in pancreatic β-cells [4]. Thus, abnormal calcium homeostasis may be associated with insulin dysfunction and glucose homeostasis, contributing to the development of T2DM [5]. Total calcium is affected by pH and serum albumin levels. Ionized calcium is not routinely tested. Therefore, serum albumin-adjusted calcium may be a better marker of ionized calcium levels [6]. On the other hand fluoride may lead to alterations in serum calcium by forming calcium fluoride [7].

## Diabetes and Mineral Homeostasis:

Diabetes significantly impacts mineral metabolism, particularly affecting calcium and fluoride levels. In individuals with diabetes, disruptions in calcium absorption and increased urinary excretion contribute to potential calcium deficiencies [8]. Additionally, excess fluoride may influence glucose metabolism [7]. Monitoring these mineral imbalances is crucial in diabetes management to prevent complications associated with deficiencies or disruptions in mineral homeostasis.

## Serum Albumin Adjusted Calcium:

Serum albumin, a major protein in blood plasma, plays a key role in calcium transport. Approximately 40% of circulating calcium is bound to albumin, forming a complex that acts as a reservoir. This bound calcium is considered biologically inactive. The remaining 60% of calcium circulates in its ionized form, which is physiologically active and readily available for cellular processes. Serum albumin acts as a carrier, facilitating the transport of calcium through the bloodstream. Changes in serum albumin levels can impact the total calcium concentration, even if the ionized calcium remains stable. Therefore, clinicians often assess both total calcium and serum albumin levels to obtain a more accurate reflection of the body's calcium status [9]. In the context of Type 2 Diabetes Mellitus (T2DM), the term "adjusted calcium" refers to a calculated value that considers the impact of alterations in serum albumin levels on total calcium concentrations. In individuals with diabetes, fluctuations in serum albumin are not uncommon either due to incipient or overt nephropathy, and since calcium largely binds to albumin, changes in albumin levels can influence total calcium readings.

The adjustment is typically made using the following formula [9]:

Adjusted Calcium (mg/dL) = Measured Total Calcium+ 0.8 x (4-Serum Albumin (g/dL)

Here, 0.8 represents the average binding capacity of albumin for calcium in grams per deci-liter.

The rationale behind this adjustment is to estimate what the total calcium concentration would be if the albumin level were within the normal range (usually considered around 4 g/dL). This correction helps in obtaining a more accurate reflection of the biologically active ionized calcium, considering the role of albumin as a major calcium carrier.

In T2DM, managing calcium levels becomes significant due to its association with various complications, including cardiovascular issues and bone health. Adjusted calcium values provide a more nuanced understanding, particularly when serum albumin levels are outside the normal range, offering clinicians a better assessment of the patient's true calcium status in the presence of diabetic conditions [10].

## Fluoride in T2DM:

Fluoride metabolism involves the absorption, distribution, and elimination of fluoride in the body. Ingested fluoride is primarily absorbed in the stomach and small intestine, with the majority accumulating in calcified tissues such as bones and teeth. Kidneys play a crucial role in fluoride excretion through urine. The concentration of fluoride in different body compartments is dynamic and influenced by various factors, including dietary intake and renal function [11].

## Potential effects of fluoride on diabetic patients:

In diabetic patients, fluoride metabolism may undergo alterations. Diabetes can affect renal function, potentially impacting the excretion of fluoride. Furthermore, diabetic individuals may experience changes in bone metabolism and composition [12]. The interplay between fluoride and diabetic conditions is complex, and studies suggest that diabetes may influence how the body handles fluoride, leading to variations in fluoride levels.

## Link between fluoride exposure and T2DM complications:

Emerging research explores the link between chronic fluoride exposure and complications associated with Type 2 Diabetes Mellitus (T2DM). Excessive fluoride intake has been investigated for its potential role in exacerbating complications related to diabetes, including impaired glucose metabolism, oxidative stress, and inflammation. The interaction between fluoride exposure and T2DM complications is an evolving area of study, seeking to understand the molecular mechanisms underlying these associations. Understanding fluoride metabolism in the context of diabetes is crucial for assessing potential health implications. It involves considering how altered fluoride dynamics may contribute to, or be influenced by, the complexities of diabetes and its associated complications [12,13].

## Factors Influencing Serum Albumin Levels:

**Figure 1:**
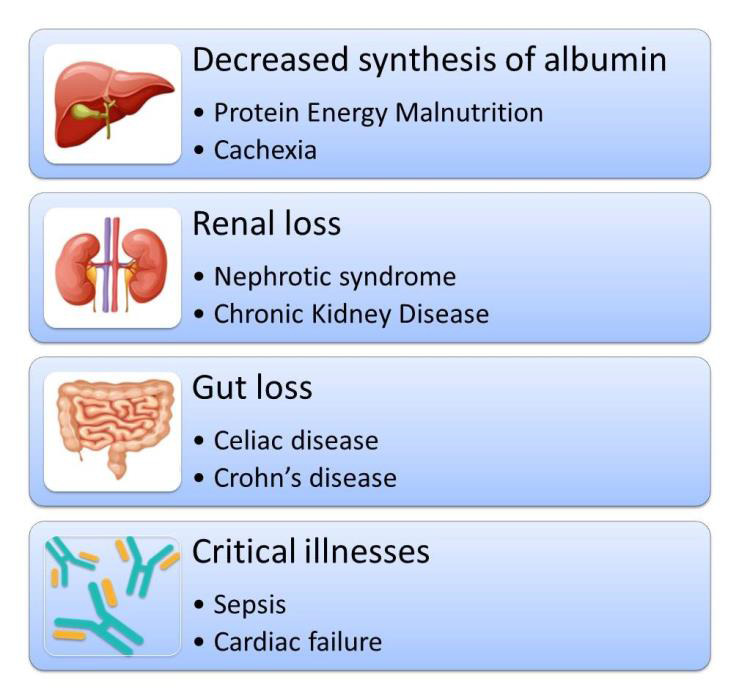
Factors affecting albumin levels. This is created using assets from http://www.freepik.com/

Serum albumin, a major calcium-binding protein, plays a crucial role in calcium transport. Understanding factors influencing serum albumin levels is vital for accurate calcium readings as depicted in Figure 1. Understanding these factors is crucial for interpreting albumin-adjusted calcium accurately and ensuring that variations in serum albumin are appropriately accounted for in clinical assessments.

## Factors Affecting Fluoride Concentrations:

**Figure 2:**
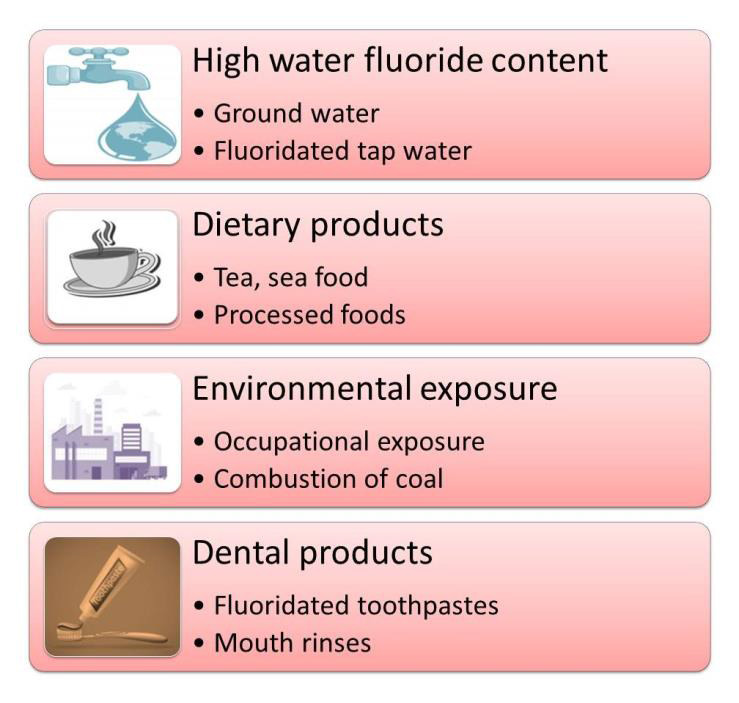
Factors affecting fluoride concentrations. This is created using assets from http://www.freepik.com/

Fluoride levels are influenced by various factors as shown in Figure 2, and understanding these essential for a comprehensive assessment.

## Methods of Measurement:

Laboratory techniques are essential for accurate assessments of serum calcium, considering albumin adjustments, and for quantifying fluoride levels in biological samples.

## Serum Albumin Adjusted Calcium quantification:

## Colorimetry:

A common laboratory technique involves the colorimetric determination of serum calcium serum albumin. This method relies on the formation of a colored complex between serum calcium and serum albumin with their specific reagents viz., Arsenazo III and Bromocresol green dyes respectively. The intensity of the color formed is directly proportional to their concentrations and can be measured colorimetrically [14,15]. To adjust calcium levels for variations in albumin, the "Albumin-adjusted Calcium" or "Corrected Calcium" formula is applied.

## Ion-Selective Electrode (ISE):

ISE is another technique for measuring serum ionized calcium. An ion-selective electrode selectively measures the concentration of calcium ions in a solution. It is particularly useful in determining ionized calcium, which represents the physiologically active form of calcium in the blood [16].

## Automated Chemistry Analyzers:

Modern clinical laboratories often utilize automated chemistry analyzers that employ various methods, including colorimetry, spectrophotometry, or ISE. These analyzers provide rapid and accurate measurements of serum calcium and albumin levels.

## Quantifying Fluoride Levels in Biological Samples

## Ion-Selective Electrode (ISE):

ISE is commonly employed to measure fluoride ion concentrations. A fluoride-selective electrode selectively responds to fluoride ions in a sample, generating a measurable electrical potential. This electrode provides a direct measurement of fluoride levels in a sample [17].

## Artificial intelligence for assessing serum calcium and fluoride status:

Artificial Intelligence (AI) plays a pivotal role in optimizing the assessment of patient calcium and fluoride status. By seamlessly integrating various patient data, such as total calcium and serum albumin, AI ensures a comprehensive dataset for accurate calculations. Integration with electronic health records (EHRs) enables real-time access to patient information; facilitating prompt adjustments based on dynamic health conditions [18,19]. These advancements underscore the transformative impact of AI in refining the precision and responsiveness of patient calcium and fluoride status assessments, ushering in a new era of personalized healthcare.

In the pursuit of establishing a reliable and easily accessible marker for calcium status across diverse hospital scenarios, this narrative review incorporates findings from a study by Bancal *et al*. The study introduces a machine learning model designed to enhance accuracy in assessing ionized calcium and, consequently, true calcium status. Utilizing total blood calcium, blood albumin, blood phosphorus, and age as predictors, the model achieved an overall correct classification rate of 78%, successfully identifying hypocalcemic, normocalcemic, or hypercalcemic states with a probability exceeding 75%. Notably, the machine learning algorithm provides results accompanied by a confidence index, allowing for a nuanced interpretation. By setting a threshold of 70%, the study aimed to balance prediction reliability and sample acceptance, yielding an 81% overall concordance rate with each calcium status correctly identified at a probability of 80% or higher [18].

**Figure 3:**
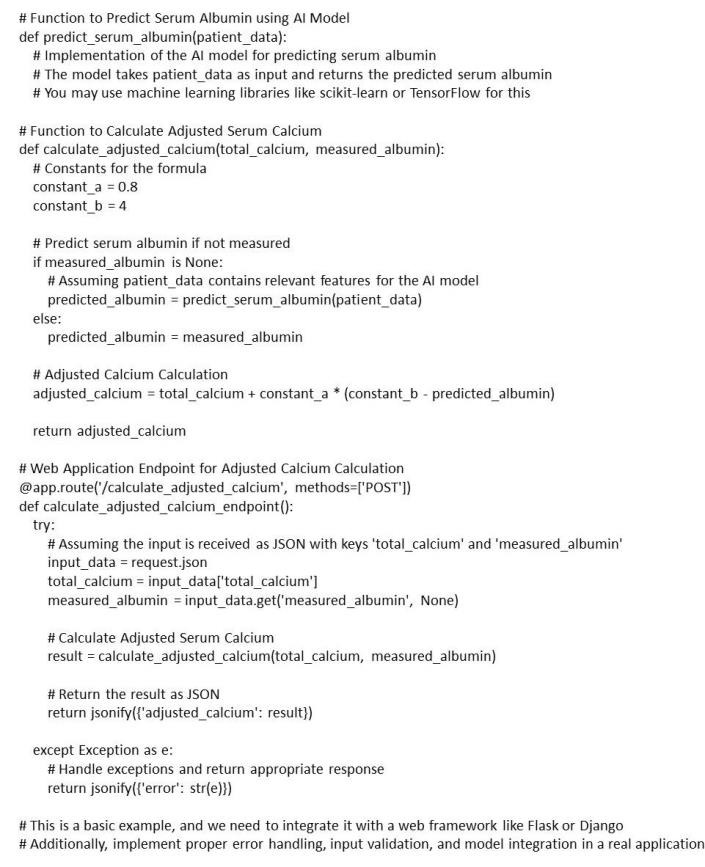
Basic structure for integrating AI model for calculating albumin adjusted serum calcium using a web application

In a research conducted by Yan *et al*. an innovative approach employing an Artificial Intelligence (AI)-integrated handheld determination platform was explored for the real-time quantitation of fluoride ions (F-) on-site. This platform utilized a 3D printed accessory and a test strip incorporating Al3+-triggered aggregation-induced red-emission enhanced carbon dots (CDs). The system was complemented by a smartphone equipped with a YOLO v3 AI algorithm-based application developed by Yan L *et al*. The feasibility of this intelligent setup for F- quantitation was demonstrated by monitoring consecutive fluorescence (FL) color changes. The carbon dots (CDs), synthesized hydrothermally from alizarin carmine and citric acid, exhibited dual emission characteristics: a moderate green emission at 512 nm and a weak red emission at 620nm under 365nm excitation. The CDs@Al3+, created by introducing Al3+ to the CDs solution, displayed distinct aggregation-induced red-emission enhancement and green-emission quenching. Additionally, the AI-integrated smartphone-based handheld detection platform proved capable of intelligent, rapid, and straightforward analysis of F- content in diverse substances such as tap water, toothpaste, and milk [19]. A basic structure for integrating AI model for calculating Albumin adjusted Serum Calcium using a Web Application is depicted in Figure 3.

## Emerging Research on the cellular mechanisms of calcium and fluoride interactions:

The intricate regulation of calcium homeostasis involves key players such as parathyroid hormone (PTH), PTH-related peptide (PTHrP), and the calcium-sensing receptor (CaSR). Wang Y *et al*., investigated the impact of fluoride on the expression of these components both in vitro and in vivo. Employing MC3T3-E1 cells and Sprague-Dawley rats, varying fluoride concentrations were administered, and free calcium ion concentrations in cell culture supernatant and serum were quantified. Low fluoride doses increased ionized calcium (i[Ca(2+)]) in cell culture supernatant, while high doses exhibited a decrease. Fluoride's influence on PTH, PTHrP, and CaSR expression was assessed using qRT-PCR and Western blot techniques. Results indicated that low fluoride doses suppressed PTH and PTHrP expression in MC3T3-E1 cells, while high doses enhanced PTHrP expression. Notably, NaF decreased serum i[Ca(2+)] in rats. Fluoride consistently up-regulated CaSR expression at both mRNA and protein levels in MC3T3-E1 cells and rats. Furthermore, fluoride exhibited differential effects on PTHrP protein expression based on the rats' diet, inhibiting it in those on a regular diet and increasing it in those on a low-calcium diet. Fluoride also induced the expression of PTH, NF-kappaB ligand (RANKL), and osteoprotegerin (OPG) in rats, with a significant increase in the RANKL/OPG ratio observed in rats on a low-calcium diet, both in the presence and absence of fluoride [20]. These findings underscore fluoride's potential to influence calcium homeostasis by modulating PTH, PTHrP, and CaSR.

## Conclusion:

The global rise in diabetes necessitates not only a cure but effective interventions for complications. The role of mineral metabolism pathways in diabetic complications remain incompletely understood and some pathways contributing to these complications may also play a role in diabetes genesis. Monitoring the serum albumin adjusted calcium and fluoride levels offers insights into risk assessment. The role of fluoride in diabetes gains attention, potentially involved in systemic metabolic processes. Incorporating assessments into diabetes care offers nuanced insights for personalized approaches. Further research is vital for elucidating intricate mechanisms and establishing evidence-based guidelines.
